# A quantitative comparison of regional myocardial motion in mice, rabbits and humans using in-vivo phase contrast CMR

**DOI:** 10.1186/1532-429X-14-87

**Published:** 2012-12-27

**Authors:** Bernd Jung, Katja E Odening, Erica Dall’Armellina, Daniela Föll, Marius Menza, Michael Markl, Jürgen E Schneider

**Affiliations:** 1 Department of Radiology, Medical Physics, University Medical Center, Freiburg, Germany; 2 Department of Cardiology, University Medical Center, Freiburg, Germany; 3 Department of Cardiovascular Medicine, University of Oxford, Oxford, UK; 4 Department of Radiology, Northwestern University, Chicago, IL, USA

**Keywords:** Cardiovascular MR, Left ventricular function, Mice, Rabbits, Humans

## Abstract

**Background:**

Genetically manipulated animals like mice or rabbits play an important role in the exploration of human cardiovascular diseases. It is therefore important to identify animal models that closely mimic physiological and pathological human cardiac function.

**Methods:**

In-vivo phase contrast cardiovascular magnetic resonance (CMR) was used to measure regional three-directional left ventricular myocardial motion with high temporal resolution in mice (N=18), rabbits (N=8), and humans (N=20). Radial, long-axis, and rotational myocardial velocities were acquired in left ventricular basal, mid-ventricular, and apical short-axis locations.

**Results:**

Regional analysis revealed different patterns of motion: 1) In humans and rabbits, the apex showed slower radial velocities compared to the base. 2) Significant differences within species were seen in the pattern of long-axis motion. Long-axis velocities during systole were fairly homogeneously distributed in mice, whereas humans showed a dominant component in the lateral wall and rabbits in the base. 3) Rotational velocities and twist showed the most distinct patterns in both temporal evolution and relative contribution of base, mid-ventricle and apex, respectively. Interestingly, a marked difference in rotational behavior during early-systole was found in mice, which exhibited clockwise rotation in all slice locations compared to counter-clockwise rotation in rabbits and humans.

**Conclusions:**

Phase contrast CMR revealed subtle, but significantly different regional myocardial motion patterns in mice, rabbits and humans. This finding has to be considered when investigating myocardial motion pattern in small animal models of heart disease.

## Background

The use of normal, surgically and genetically manipulated small animal models for basic cardiac research is based on three fundamental assumptions: (a) mechanics and function of the heart are comparable across species under baseline conditions; (b) disease models have to be informative the same phenotype across models; (c) one can project from one to another species, in spite of differences, as long as one knows those differences. Surgical models such as transient or permanent ligation of the left coronary artery [1] and aortic constriction [2] are available to mimic acute or chronic myocardial infarction, and hypertrophic cardiomyopathy / heart failure. Conversely, in genetically manipulated animals the genome has been modified to reproduce molecular causes of heart disease [3]. The ultimate aim is to create structural and functional conditions found in patients in order to understand (patho-) physiological pathways and molecular mechanisms of diseases to ultimately develop novel treatments. For practical reasons, i.e., due to their small size and low housing costs compared to larger mammalians, their short reproduction times, and their well-defined genome, mice have become a species of choice in cardiovascular research. More recently, transgenic rabbit models have been introduced as animal models that more closely mimic the human cardiovascular physiology, and therefore, are also used to explore molecular causes of human cardiovascular diseases [4-6].

However, the similarity in cardiovascular physiology and functional response to genetic manipulation implies an affinity in mechanical myocardial function between species. There are in fact dramatic differences not only in size, hemodynamic parameters (such as heart rate, blood pressure and ejection fraction), but also in the myocardial fibre orientation throughout the ventricular wall [7-10] that may contribute to species differences in mechanical myocardial function. Furthermore, species differences in the relative long-axis (also termed as longitudinal) contribution to systolic ventricular function [11] and at the structural sarcomeric level [12] as well as different types of ion channels have been described [13], resulting in pronounced differences in duration and shape of action potential in mice as compared to rabbits and humans [14]. Based on the close electro-mechanical coupling, differences in ion currents and action potential duration may also translate into differences in electromechanical function. The use of different transgenic animal models in basic cardiovascular research thus prompts a detailed investigation of cardiac mechanics on a regional level of the **normal** animal heart compared to the healthy human heart to identify animal models that closely mimic physiological human cardiac function, before using the most suitable models to investigate pathological cardiac function.

Traditional ECG gated and time-resolved (Cine) CMR enables the evaluation of the global left ventricular (LV) function by assessing myocardial wall thickening and ejection fraction in humans [15] as well as in mice [16] and rabbits [17,18]. Phase contrast CMR or tissue phase mapping (TPM) is a well-established technique providing high spatial and temporal resolution for an accurate and reliable quantification of the regional cardiac function by encoding the velocity in the phase information of the MR signal [19]. Myocardial velocities are sensitive markers of myocardial contractility [20] and active relaxation [21] of the myocardium. During the early stage of hypertensive heart disease, i.e. with preserved ejection fraction, it has been shown that velocities are reduced before global strain or global function are affected [22]. TPM is readily available in humans [19] and mice [23-25], and allows for a comprehensive regional analysis of the three-directional – radial, long-axis, rotational (also termed as circumferential) – ventricular velocities. However, a direct comparison of a comprehensive segmental cardiac function between different animal models and humans is lacking.

The aim of this study therefore was to apply TPM in mouse, rabbit, and human hearts in order to evaluate potential differences in regional LV function. This characterization and understanding of regional cardiac function across species under baseline conditions will be of importance to identify and correctly interpret the functional consequences of any intervention and, ultimately, treatment.

## Methods

### MR hardware

A 1.5T MR-system was used for the TPM measurements on humans (Sonata, Siemens, Germany) and rabbits (Avanto, Siemens, Germany). Human scans were performed with a 12-channel thorax coil and rabbit scans with a 12-channel head coil. Murine measurements were performed on a 9.4T VNMRS DirectDrive MR-system (Varian Inc, USA) using a quadrature-driven birdcage resonator (Rapid Biomedical, Rimpar, Germany).

### Human study

Twenty healthy volunteers (mean age 30±4 years) without history, symptoms, or medication of cardiovascular disease were enrolled in the study. Written informed consent was obtained from all volunteers, and the study approved by our local ethics board.

### Animal preparation

C57Bl/6 mice (n=18) were obtained from a commercial breeder (Harlan, UK) at least one week prior to the imaging time point to allow naturalization to new surroundings. Anesthesia was induced in an anesthetic chamber using 4% isoflurane in 100% oxygen. Animals were then positioned prone on dedicated mouse cradles and maintained at 1.5-2% isoflurane at 2 l/min oxygen flow throughout the CMR experiments. A 1 ml tube containing 1% agarose and 2 mM Gd-DTPA was placed next to the chest of the animal to correct for background phase errors (the correctness of sequence and post-processing was validated in phantom experiments prior to the in-vivo application). Temperature was maintained at ~37°C using a warm air blanket placed below the animal. A thermocouple is always placed inside the heating blanket providing feedback to the temperature control unit, and thus maintaining physiological conditions for the animal. Cardiac and respiratory signals were continuously monitored and used for a combined ECG- and respiratory gating.

New Zealand White rabbits (200±14 days, n=8) from in-house breeding colonies were used for experiments. Animals were anesthetized with ketamine/xylazine IM (12.5 mg/kg/3.5 mg/kg), shaved, and positioned on their back. Temperature was maintained by placing blankets underneath the animal. Anesthesia was maintained with ketamine/xylazine IV (1–2.5 ml/kg/h) and 4 l/min oxygen flow via mask throughout the CMR experiments. Cardiac signals were obtained with ECG electrodes and devices as used for human measurements.

All animal studies were performed in accordance with the local institutional guidelines and only after approval by the Regional Board, the Institutional Animal Care and Use Committee in accordance with the *Guide for the Care and Use of Laboratory Animals* published by the U.S. National Institutes of Health (NIH Publication No.85-23, revised 1996), or the Home Office *Guidance on the Operation of the Animals (Scientific Procedures) Act*, 1986 (HMSO).

### TPM imaging

**Figure 1:**
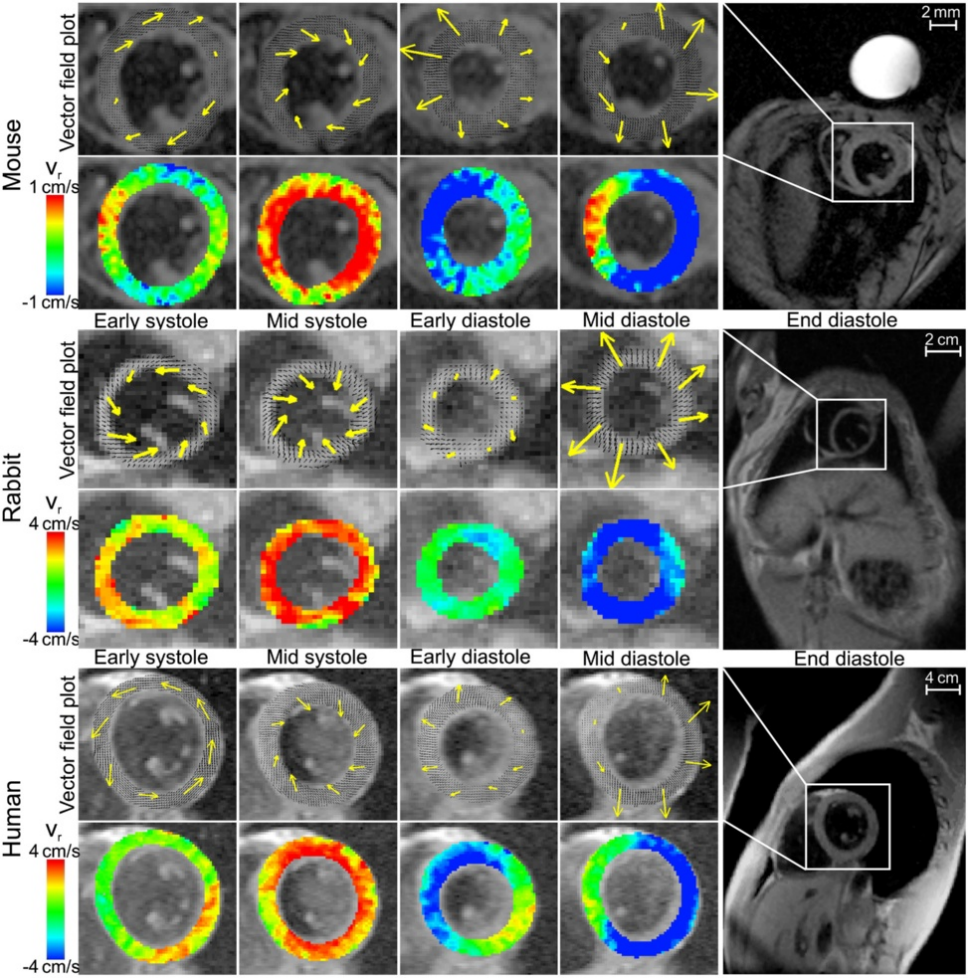
**Pixel-by-pixel vector field plots and color-coded maps of radial velocities of in-plane velocities (red: contraction, blue: expansion) in a basal slice for four characteristic cardiac frames.** Note the different rotational behavior during early systole in the vector field plots for the mouse compared to the human subject and the rabbit. The yellow arrows are included for better visualization of the vector field and are determined by averaging the pixel-wise vectors within each of eight myocardial regions.

All experiments were performed with a Cine gradient echo sequence with prospective ECG-gating using a black blood preparation module to suppress blood flow related artifacts. Measurements on volunteers were performed with navigator gating allowing for acquisition during free breathing [26], while a respiratory gating device was applied for measurements in mice [27]. No respiration control was necessary for rabbits due to a relatively shallow breathing without significant cranio-caudal cardiac displacement under anesthesia. All scan parameters are summarized in Table 1. A slightly lower spatial resolution relative to the LV size was obtained for rabbits (as can be observed in Figure 1) due to signal-to-noise limitations at 1.5T.

**Table 1:** **CMR parameters for mice**, **rabbits and human measurements**

	**Mice**	**Rabbits**	**Humans**
MR scanner [Tesla]	9.4	1.5	1.5
Matrix size	128 × 128	160 × 98	256 × 96
Spatial resolution [mm]			
- acquired	0.2 × 0.2	1.0 × 1.2	1.3 × 2.6
- reconstructed	0.1 × 0.1	1.0 × 1.0	1.3 × 1.3
Slice thickness [mm]	1	4	8
TE / TR [ms]	2.1 / 4.6	5.4 / 7.6	5.0 / 6.9
Temporal resolution [ms]	4.6	7.6	13.8
Venc in-plane [cm/s]	6	10	15
Venc through-plane [cm/s]	8	15	25
Bandwidth [Hz/pixel]	1150	650	650
Flip angle [°]	15	15	15
Averages	2	4	1
Scan time per slice [min]	3	8	5
SNR ROI anteroseptal	29.1 ± 2.9	37.2 ± 4.8	43.7 ± 8.8
SNR ROI inferolateral	25.6 ± 4.6	32.7 ± 5.4	40.4 ± 10.3

Three short axis series (base, mid, apex) were acquired in all examinations. In all species, the basal slice was positioned on the end-systolic 4-chamber view perpendicular to the septal wall so that the slice was located in the septal wall without touching the LV outflow tract in systole. The apical slice was located to be within the blood pool of the apical region; for mice and rabbits this was one slice up towards the base from the end-systolic position of the apex, for humans two slices (corresponding to a position of about 75% of the distance between the atrioventricular groove and the apex in the horizontal diastolic long axis). The mid-ventricular slice was positioned equidistant to the basal and the apical slice.

For humans and rabbits the black blood saturation module (6 ms) was played out after the detection of the R-wave, therefore the first cardiac frame was acquired 17.9 ms for humans and 11.4 ms for rabbits after the R-wave. In our murine experiments, the black blood saturation module (7.5 ms) was applied at the end of the Cine train before detection of the next R-wave, therefore, allowing for the acquisition of the first cardiac frame 2.1 ms (TE) after the R-wave. Thus, about the last 20% of the murine cardiac cycle was not covered. However, in all subjects peak velocities were acquired. Due to the 8-10-fold higher heart rate in mice compared to humans, the sampling density in murine experiments was only about 65% compared to humans or rabbits despite the higher temporal resolution. TPM in rabbits was performed as a new study and compared with the subgroups of mice and humans from previous studies [23,28].

### Data analysis

Morphological characteristics of LV such as diastolic length (mitral valve to apex), diastolic diameter (mid of myocardium), diastolic mid-ventricular wall thickness / radius, end-diastolic and end-systolic volumes as well as LV mass and ejection fraction were determined from conventional short axis Cine images acquired over the entire LV. Additionally, approximated (due to the phased array coils) signal-to-noise-ratio (SNR) was calculated as the mean value in the region-of-interest (ROI) over the standard deviation in a ROI outside the subject in an anteroseptal and in an inferolateral ROI, respectively.

All TPM-data post-processing was performed using customized software programmed in Matlab (The Mathworks Inc, Natick, US). After manually delineating the epi- and endocardial borders on a frame-by-frame and slice-by-slice basis, the measured in-plane velocities (*v*_*x*_ and *v*_*y*_) were transferred into an internal polar coordinate system, positioned at the center of mass of the LV (of the LV segmentation mask), and expressed as radial (*v*_*r*_) and rotational (circumferential, *v*_*φ*_) velocities. The measured through-plane velocities (*v*_*z*_) reflect long-axis motion of the LV, therefore termed as long-axis velocities. Furthermore, all velocities were corrected for translational motion components, for the in-plane velocities based on subtraction of global translation velocities from the local velocity components [29], the through-plane velocity curves were shifted in order to meet the physiological condition that the integral must result to zero. Global cardiac motion patterns were analysed by averaging the velocity components over the entire segmentation mask of each individual slice, resulting in velocity time courses for basal, mid-ventricular, and apical slices. To facilitate a more detailed analysis of segmental cardiac function the LV was partitioned at each time frame into 6 equi-angular segments on basal and mid-ventricular, and 4 equi-angular segments on apical slice level based on the 17-segment model according to American Heart Association (AHA) and American College of Cardiology (ACC) recommendation [30]. The 17^th^ segment, which only contains the apex, was excluded from analysis. Mean radial (*v*_*r*_) and long-axis (*v*_*z*_) systolic and diastolic peak velocities were determined for all 16 segments. To investigate regional differences the velocity-ratios between lateral and septal wall, anterior and inferior wall, and basal and apical segments were determined as dimensionless parameter for a comparison between species (see lower right depiction in Figure 5). The twist angle Φ for each slice was calculated over time according to

(1) φt=∫1tvϕt'rt'dt'

where *r* is the middle ((r_epi_-r_endo_)/2) LV radius at the time frame *t*[31]. This allowed for calculating the ventricular net twist angle i.e. the torsion, Φ_Torsion_ = Φ_Apex_ – Φ_Base_. Furthermore, twist angle and torsion were also assessed for the epi- and endocardial layers based on the respective middle radius.

**Figure 2:**
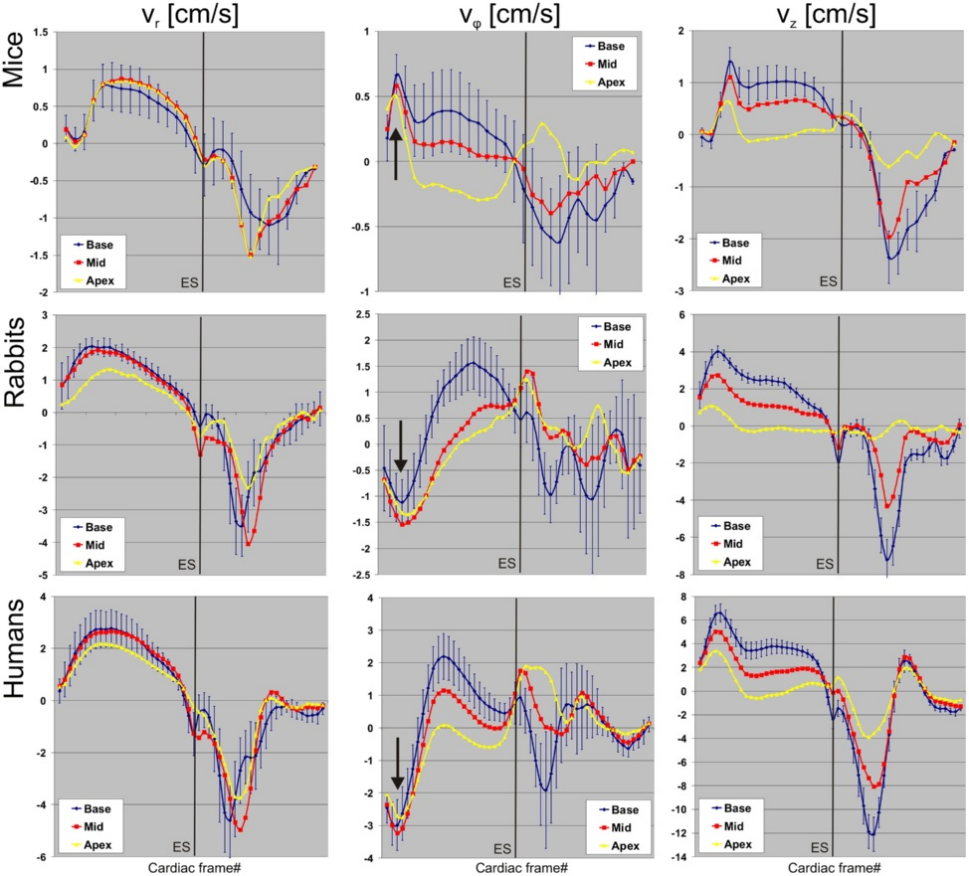
**Averaged global velocity time courses for the three different velocity components (columns) and the three different species (rows).** The systole of the time courses was normalized as indicated by end-systole (ES). For better visualization, standard deviations are only shown for the basal slice but were comparable for mid and apical slices. Note the different evolution of rotational velocities during early systole between species (see arrows).

To avoid temporal jitter, all velocity time curves were normalized by interpolating the systole to the same number of frames. For this purpose, the end-systolic time was defined by the first minimum peak of the global radial velocities during diastole (isovolumetric relaxation, see Figure 2), which could be observed in all measurements. The diastole was not normalized. As previously described [19], the sign-convention for the LV velocities used throughout the paper was as follows: a positive radial velocity indicates contraction, i.e. the myocardium moves towards the centre-of-mass. A positive in-plane rotation is defined as clockwise when *viewed from apex to base*. The long-axis velocity is considered positive if the myocardium moves from base to apex (systolic shortening).

**Figure 3:**
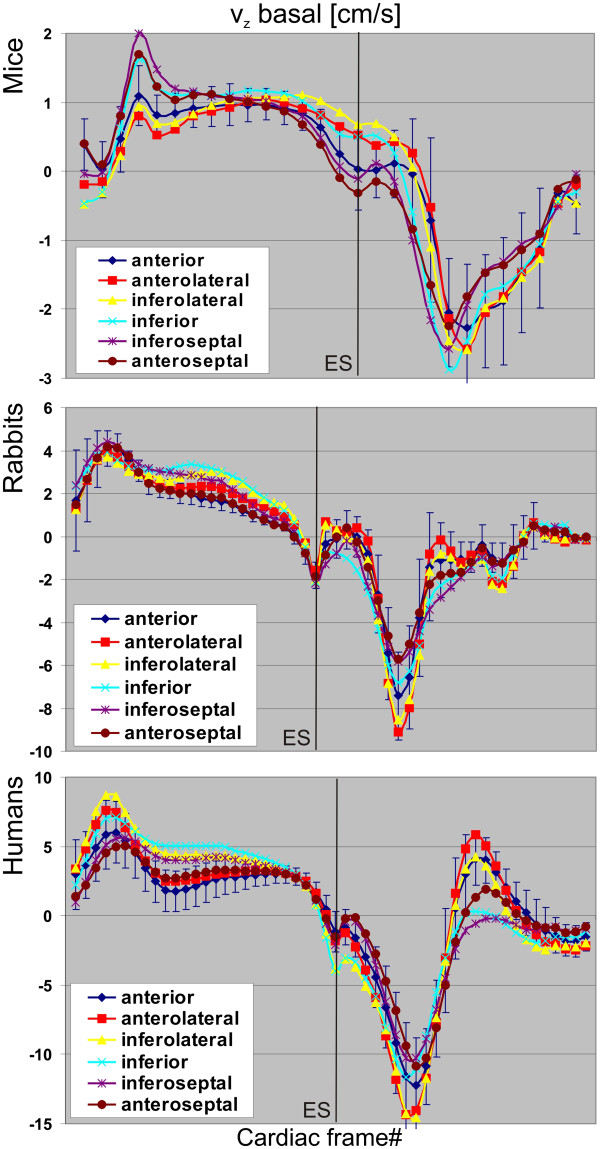
**Averaged segmental velocity time courses for basal long-axis velocities for the three different species.** See text for more information. Standard deviations are only shown exemplarily for the anterior segment due to visualization purposes.

All data are presented as mean ± SD. The differences between the three species were analyzed using a 1-way ANOVA followed by a Bonferroni test for pairwise multiple comparisons using SPSS (IBM, USA). Comparisons between epi- and endocardial twist were performed by a two-tailed paired t-test. A p<0.05 was considered statistically significant.

## Results

Mean body weights, heart rates, LV morphological characteristics and ejection fractions are summarized in Table 2. The ratio of myocardial mass and volumes between rabbits and mice (~50-100) is 2–4 times higher compared to the ratio between humans and rabbits (~25). Noticeably, wall thickness normalized to LV radius was significantly increased in mice compared to rabbits (p<0.001) and humans (p<0.001), while the latter two revealed no significant differences. Note the low standard deviations for the wall thickness to radius ratio in all species. A significantly larger indexed LV-Mass (i.e. LV-mass/bodyweight) was found for mice (3.24 ± 0.27 vs. 1.13 ± 0.18 (rabbits) or 1.38 ± 0.27 (humans), p<0.001).

**Table 2:** **Body weights**, **heart rates**, **morphological characteristics of LV and ejection fraction**

	**Mice**	**Rabbits**	**Humans**
Number of subjects	18	8	20
Body weight [kg]	0.023 ± 0.0017	4.0 ± 0.43	67.2 ± 12.1
Heart rate [bpm]	464 ± 35	186 ± 30	65 ± 6
Length [mm]	8.1 ± 0.4	32.3 ± 1.2	97.9 ± 9.9
Diameter [mm]	5.5 ± 0.2	22.7 ± 0.9	69.6 ± 5.2
Wall Thickness [mm]	0.80 ± 0.04	2.4 ± 0.1	7.1 ± 0.7
Wall Thickness / Radius	0.29 ± 0.01	0.21 ± 0.01	0.20 ± 0.01
LV-Mass [g]	0.074 ± 0.005	4.05 ± 0.21	104.4 ± 15.8
EDV [ml]	0.046 ± 0.007	4.61 ± 1.33	126.1 ± 16.4
ESV [ml]	0.012 ± 0.005	1.88 ± 0.50	57.1 ± 7.9
SV [ml]	0.034 ± 0.005	2.74 ± 0.76	69.9 ± 9.0
Ejection Fraction [%]	73.4 ± 8.3	59.2 ± 5.3	54.8 ± 2.5
LV-Mass_indexed_ [×1e-3]	3.24 ± 0.27	1.13 ± 0.18	1.38 ± 0.27
EDV_indexed_ [ml/kg]	2.0 ± 0.31	1.42 ± 0.25	1.56 ± 0.41
ESV_indexed_ [ml/kg]	0.56 ± 0.23	0.56 ± 0.10	0.67 ± 0.22
SV_indexed_ [ml/kg]	1.44 ± 0.20	0.86 ± 0.18	0.89 ± 0.21

The indexed parameters are normalized to the body weight. (Mean ± SD).

Figure 1 shows examples for velocity vector field plots displaying the in-plane velocities and color-coded maps of radial velocities in a basal slice at four characteristic time frames during the cardiac cycle of an individual mouse (top rows), rabbit (mid rows) and human subject (bottom rows) demonstrating the overall image quality of the different species measurements. In rabbit and human, rotational behavior during early (isovolumetric) systole is bi-phasic, showing an initial short positive twisting, followed by a more pronounced and extended period of negative rotation. In mouse, the initial counter-rotation is absent. A similar behavior of the rotational and radial motion evolved during mid-systole for all species.

The global velocity time courses for the three different velocity components (columns) and the three different species (rows) are summarized in Figure 2, where each panel illustrates the information from the three acquired slices. Figure 3 displays representative examples of mean segmental velocity time courses showing basal long-axis velocities for all species. Mentionable, the velocities in the first cardiac frame do not start from zero values since data acquisition do not coincide exactly with the R-wave. The most important differences of myocardial velocities between mice, rabbits and humans that can be observed in the graphs of Figures 2 and 3 (not all shown as in Figure 2) are as follows:

**Figure 4:**
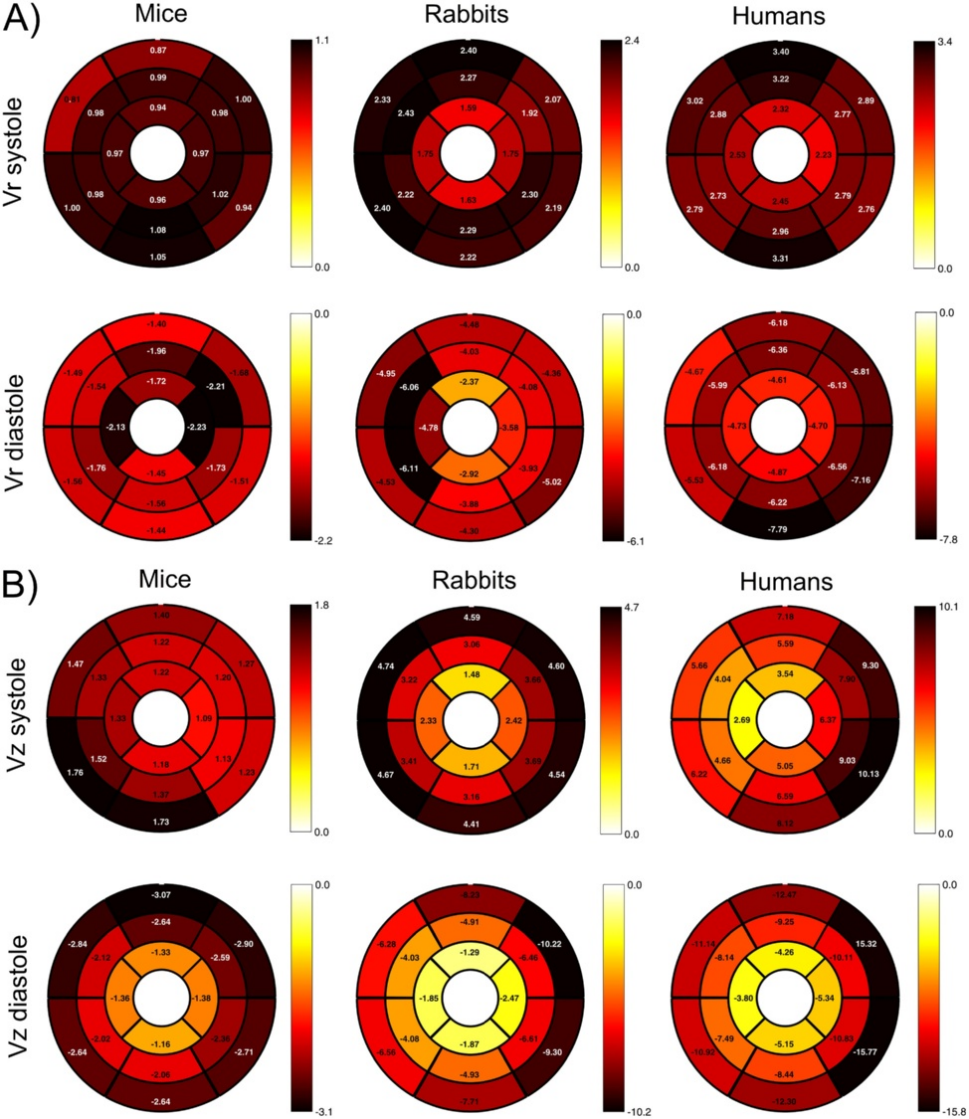
**Bulls-eye plots for systolic and diastolic radial (*v***_***r***_**, A) and long-axis (*v***_***z***_**, B) peak velocities (mean values of all animals) within the 16 AHA-segments of the three different species.** Radial velocities (**A**) show similar regional distributions of peak velocities whereas more pronounced differences are found for long-axis peak velocities (**B**) such as more homogenously distributed systolic murine velocities and lower velocities for apical regions in rabbits and humans compared to basal segments.

*Global velocity time courses (Figure*2*):*

• Systolic *v*_*φ*_: clockwise rotation during early systole in mice, counter-clockwise in rabbits and humans (all slices, see arrows). Almost constant velocities after initial clockwise rotation in mice, pronounced increase of velocities in rabbits and humans (all slices).

• Diastolic *v*_*φ*_: initial clockwise rotation in all slices in rabbits and humans, in mice only observable in apical slice.

• Systolic *v*_*r*_: highest velocities in mid-ventricular and apical slices in mice, in basal and mid-ventricular in rabbits and humans.

• Diastolic *v*_*r*_: earliest maximal velocities in base for rabbits and humans, for mice in apical and mid-ventricular slices, basal slice clearly delayed. Highest peak velocities for basal and mid-ventricular slices for rabbits and humans, for mice in apical and mid-ventricular slices.

• Diastolic *v*_*z*_: negative dip during isovolumetric relaxation more pronounced in rabbits compared to humans, not observable in mice (all slices).

Segmental velocity time courses:

• Highest velocities in lateral wall in mice, in septal wall in rabbits, humans demonstrated a more homogenous distribution of peak velocities (mid-ventricular slice).

• Systolic *v*_*z*_: highest velocities in lateral wall in humans, in septal wall in mice, and more homogenously distributed in rabbits (all slices).

• Diastolic *v*_*z*_: highest velocities in lateral wall in rabbits and humans, more homogenously distributed in mice (all slices).

The averaged radial and long-axis peak velocities during systole and diastole as well as the rotational peak velocities during early and mid systole are summarized in Table 3. Peak velocities in rabbits were significantly higher compared to mice (p<0.001) for all velocity components (systole and diastole) and slices. Furthermore, peak velocities in humans were significantly higher compared to rabbits (p<0.005) for all velocity components except for radial diastolic velocities in all slices and apical rotational mid-systolic velocities (p=ns). Differences in peak velocities between mice and rabbits (reduction of *v*_*r*_ and *v*_*z*_ by a factor of 2–3) seem to be more pronounced than the differences between rabbits and humans (reduction of *v*_*r*_ and *v*_*z*_ by 20–40%). The most pronounced differences were found in rotational velocities: peak velocities during early systole in mice were smaller by a factor of 5 and additionally reversed (opposite rotation direction) compared to humans (Figure 2). During mid systole, rotational peak velocities were oriented along the same direction for both mice and humans but values in mice were clearly smaller. In contrast, TPM analyses in rabbits revealed a more similar rotational behavior compared to humans with similar rotation direction but significantly lower peak velocities (p<0.001), i.e. a factor of 2–3 during early systole and by 1.5 during mid systole. Higher systolic and diastolic peak velocities (*v*_*r*_ and *v*_*z*_) were observed in the basal slice compared to the apical slice for humans and rabbits, while there were only minor non-significant differences between base and apex in mice. Note the relatively low standard deviations of peak velocities representing small intra-species differences.

**Table 3:** **Global peak velocities in murine**, **rabbit and human hearts** (***v***_***r***_: **radial, *v***_***φ ***_: **rotational, *v***_***z***_: **long**-**axis**)

**Velocity**	**Slice**	**Peak Systolic velocity** [**cm**/**s**]			**Peak Diastolic velocity** [**cm**/**s**]		
		**Mice**	**Rabbits**	**Humans**	**Mice**	**Rabbits**	**Humans**
*v*_*r*_	basal	0.9 ± 0.2	2.1 ± 0.2	2.9 ± 0.7	−1.4 ± 0.3	−4.3 ± 0.4	−5.2 ± 1.4
	mid	0.9 ± 0.2	2.0 ± 0.4	2.7 ± 0.7	−1.6 ± 0.5	−4.5 ± 0.8	−5.3 ± 1.5
	apical	0.9 ± 0.2	1.4 ± 0.2	2.3 ± 0.7	−1.8 ± 0.4	−3.3 ± 0.7	−4.4 ± 1.8
*v*_*z*_	basal	1.4 ± 0.4	4.3 ± 0.9	7.4 ± 1.5	−2.7 ± 0.4	−8.0 ± 1.0	−12.7 ± 2.9
	mid	1.2 ± 0.3	3.2 ± 0.7	5.8 ± 1.5	−2.3 ± 0.6	−4.9 ± 1.3	−8.7 ± 2.7
	apical	0.9 ± 0.3	1.6 ± 0.5	4.0 ± 1.4	−1.1 ± 0.6	−1.3 ± 0.5	−4.3 ± 1.7
		**Peak early**-**systole**			**Peak mid**-**systole**		
*v*_*φ*_	basal	0.8 ± 0.4	−1.3 ± 0.5	−3.7 ± 1.8	0.1 ± 0.2	1.6 ± 0.4	2.6 ± 1.1
	mid	0.7 ± 0.3	−1.8 ± 0.5	−3.9 ± 1.7	−0.1 ± 0.1	0.9 ± 0.5	1.5 ± 1.0
	apical	0.7 ± 0.3	−1.5 ± 0.5	−3.3 ± 1.4	−0.3 ± 0.2	0.5 ± 0.6	0.5 ± 0.8

The peak velocities between all species are significantly different (p < 0.005) except for the radial diastolic velocities between rabbits and humans (all slices).

Figure 4 depicts color-coded bulls-eye plots illustrating segmental systolic and diastolic radial and long-axis peak velocities within the 16 AHA-segments of the three different species. Similar regional distributions can be observed for the radial systolic (upper row in Figure 4A) and diastolic (lower row in Figure 4A) peak velocities except for lower apical peak velocities in rabbits and humans compared to mice. More pronounced differences were observed for the long-axis peak velocities as shown in Figure 4B. Systolic murine velocities were more homogenously distributed over all LV segments, whereas lower velocities were found for more apical regions in rabbits and humans compared to basal segments.

**Figure 5:**
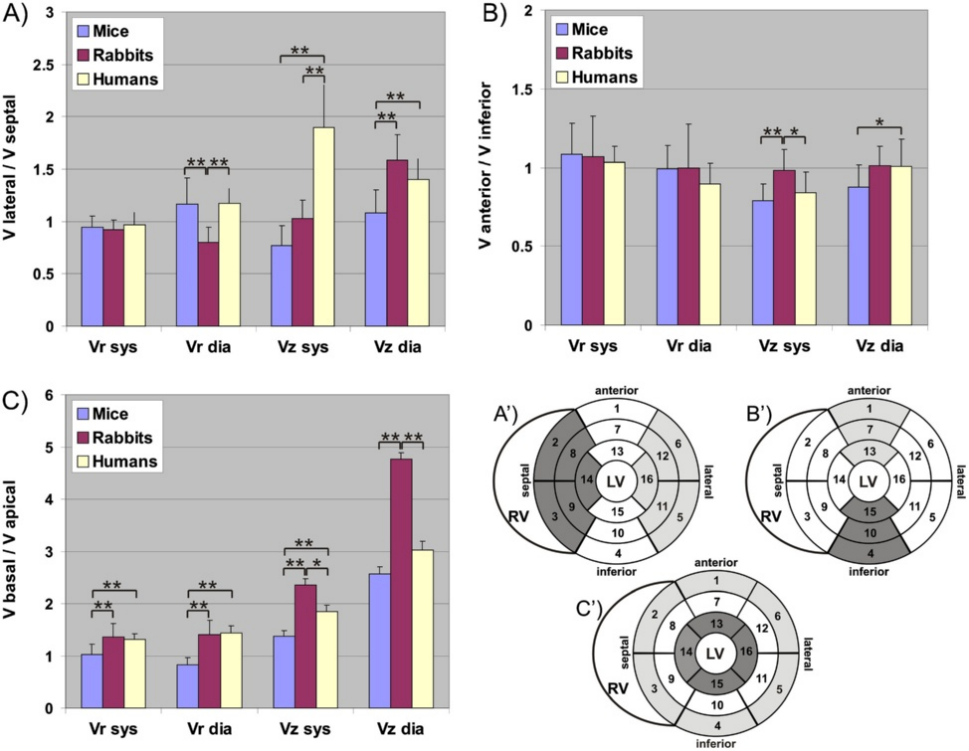
**Velocity ratios lateral/septal (A), anterior/inferior (B), and basal/apical in murine, rabbit and human hearts for radial and long-axis velocities during systole and diastole.** Significant differences between species are indicated by *(p<0.05) and **(p<0.001). **A’-C’**: pictograms of AHA 16-segment models that illustrate the regions used for ratio calculation in **A-C**, regions are highlighted in dark and bright grey.

Differences for the ratio *R* of lateral to septal peak velocities were observed (Figure 5A): *R*_*rabbits*_*< R*_*mice*_ and *R*_*rabbits*_*< R*_*humans*_ (p<0.001) for diastolic *v*_*r*_, *R*_*mice*_*< R*_*rabbits*_ (p<0.05) *< R*_*humans*_ (p<0.001) for systolic *v*_*z*_, and *R*_*mice*_*< R*_*rabbits*_ (p<0.001), *R*_*mice*_*< R*_*humans*_ (p<0.001), *R*_*rabbits*_*> R*_*humans*_ (p<0.05) for diastolic *v*_*z*_. Less pronounced differences were found when analyzing the ratio of anterior to inferior velocities (Figure 5B). Here, only a higher ratio of systolic long-axis velocities was found in rabbits compared to mice (p<0.001) and in rabbits compared to humans (p<0.05) as well as a lower ratio of diastolic long-axis velocities in mice compared to rabbits (p<0.05) and in mice compared to humans (p<0.05). Velocity distributions were confirmed by the analysis of the ratio of basal to apical peak velocities in Figure 5C revealing significant differences (p<0.001) in mice vs. rabbits and mice vs. humans for radial systolic and diastolic velocities as well as systolic long-axis velocities. The only exceptions are the diastolic long-axis velocities, where significant differences (p<0.001) were found between mice vs. rabbits and rabbits vs. humans, but not in mice vs. humans. Mice always exhibited the lowest ratio of basal to apical velocities, even with higher apical radial diastolic velocities (i.e. ratio <1).

**Figure 6:**
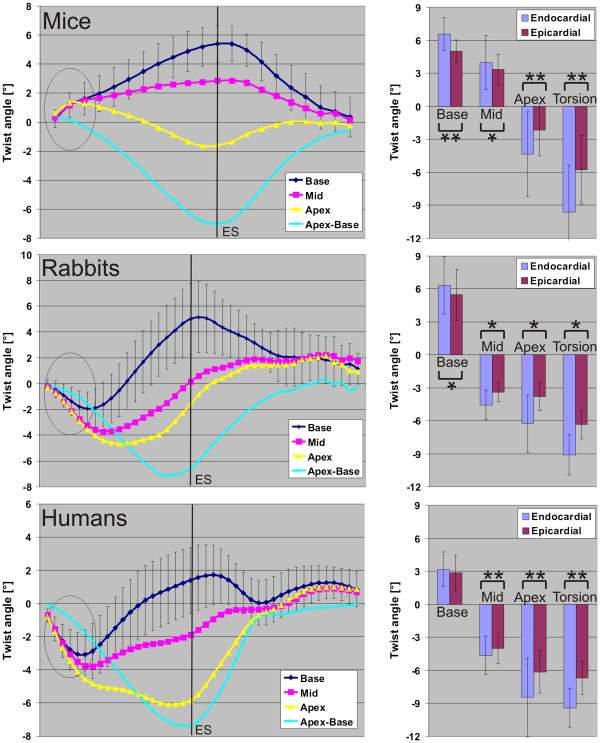
**Left graphs: Temporal evolution of the twist angle calculated from rotational velocities in all three slices and the torsion angle Φ**_**torsion **_**= Φ**_**Apex **_**– Φ**_**Base**_. Standard deviations are only shown for the basal slice for better visualization purposes but were comparable for mid and apical slices. The circles indicate the early systolic phase, which depicts opposite twists between mice and rabbits / humans. Right graphs: The basal and apical maximum twist angle and the maximum torsion angle (apex - base) for the epi- and the endocardium. Significance between compartments are indicated by * (p < 0.05) and ** (p < 0.001).

The graphs in Figure 6A-C display the evolution of the twist angle over the cardiac cycle in all slices as well as the torsion Φ_Torsion_ = Φ_Apex_ – Φ_Base_. Consistent with differences in rotational velocities in Figure 2, changes in the temporal evolution of the twist angle can be observed. The initial systolic counter-clockwise rotation of all three slices as indicated by the circles in each panel resulted in twist angles of 2–5° in both rabbits and humans. In contrast, a clockwise rotation of about 1° was found for murine twisting. Interestingly, the mid-ventricular slice in mice, unlike rabbits and humans, exhibits only positive twists throughout the cardiac cycle. Nevertheless, the mean maximum torsion was similar in all species (mice 7.6° ± 3.3°, rabbits 7.8° ± 1.3°, humans 7.9° ± 1.6°; p=ns).

**Figure 7:**
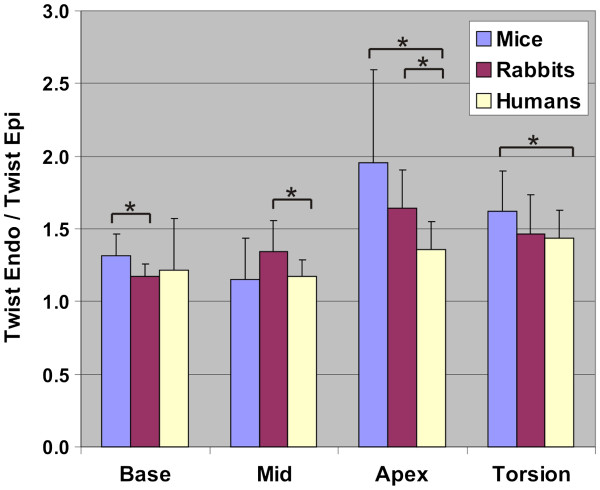
**Maximum twist angle ratios endocardial/epicardial for all slices and for the torsion.** Significance between compartments are indicated by * (p < 0.05) and ** (p < 0.001).

The right column in Figure 6A-C shows the maximum epi- and endocardial twist in all slices as well as the torsion. As expected, significant differences between compartments can be observed in all cases except for the basal twist in humans. The highest ratio between epi- and endocardial twist always appears in mice. Significant differences of the ratio between species are only present between mice and rabbits in the apical twist, between mice and humans in the basal twist and the torsion, and between rabbits and humans in the basal twist (all p<0.05). Figure 7 summarizes the ratios between endo- and epicardial maximum twist angle in all slices and for the torsion revealing a trend towards a higher ratio in mice in the apex and particularly in the torsion angle as compared to rabbits / humans.

## Discussion

This is the first study that uses TPM to assess species differences in regional myocardial function. Importantly, we here provide the first detailed assessment of normal segmental cardiac function in two small animal models, commonly used in cardiovascular research, and compare them to humans. Contrary to the fundamental assumption of similar regional myocardial function across the various animal models, our findings demonstrate that there are significant species differences already under physiological baseline conditions. Therefore, genetical or surgical interventions typically used to investigate pathological cardiac function in diseases and their underlying mechanisms could lead to different outcomes in animal models as compared to humans.

Specifically, our main findings are:

• The peak radial velocities are fairly homogeneously distributed throughout the left ventricle in mice and humans, while they are significantly higher in base and mid-ventricle in rabbits.

• The base is moving towards the apex in all species, but peak-velocities are highest in inferior / septal segments in mice, and lateral segments in humans, and homogeneously in basal segments in rabbits, respectively.

• Rotational velocities in mouse hearts are positive at early systole (indicating clockwise rotation when viewed from apex to base) but negative in rabbits and humans (i.e. counter-clockwise rotation).

• The torsion (i.e. the net twist) between base and apex is similar across all species. However, the temporal evolution and relative contributions between base and apex varies within species.

Importantly, we are first to demonstrate significant differences between mice and humans in the early systolic rotational motion of the LV and to provide a comprehensive comparison of segmental motion. Two tagging studies were published so far comparing myocardial motion across species. Henson et al. reported on the LV twist in mice and humans [32] with a low temporal resolution (9.4 and 42 ms for mice and humans) and a low spatial tag resolution (1.2 and 7 mm for mice and humans) only demonstrating the same rotational behavior as a sum over the systole. Furthermore, only ventricular twist and strain were quantified without performing a segmental analysis. Liu et al. performed a comparison between mice, rats, and humans [33] but with a relatively low temporal resolution (8, 14, and 37 ms for mice, rats, and humans) compared to the time scale of myocardial motion, and with low spatial tag resolution (0.6, 0.9, and 6 mm for mice, rats, and humans, respectively). Moreover, only ventricular twist and strain were quantified without a segmental analysis. Both tagging studies [32,33] confirmed the same opposite rotation of apex and base during mid systole as we report across species, e.g. with a systolic clockwise net twist of the base and a counter-clockwise twist of the apex when viewed from apex to base. The net twist angle between base and apex in our study was similar in mice, rabbits and humans (~8°, with somewhat higher standard deviations in mice) as recently reported by Zhou et al. [34].

As mentioned above, the global LV counter-clockwise rotation during early systole in humans and rabbits contrasting with a global clockwise rotation in mice observed in our study, however, has not yet been reported to date. This is likely due to the fact that in the mice studies from Henson and Liu A) the tagging module (7–10 ms) was played out after the detection of the R-wave and B) the temporal resolution (8–9.4 ms) was much lower than in our study (4.6 ms; with the acquisition of the first cardiac frame 2.1 ms (TE) after the R-wave) thereby causing the absence of the first three cardiac frames that demonstrate this different rotational behaviour during early systole in our mice measurements. Furthermore, the lower spatial resolution provided by the tag grid pattern may limit the detection of these subtle short-range differences in the rotational motion pattern [32,33]. Despite the same limitations (the tagging module after the R-wave and the low temporal resolution of 8–10 ms), a similar twist behaviour in all slices (as shown in Figure 6) can also be observed in the data by Li et al. [35].

More significant regional differences between species were detected that have not been reported so far. Most pronounced segmental differences occurred for the long-axis velocities as evidenced by Figure 5. Noticeably, the lateral-septal difference for the systolic long-axis velocities was not visible in rabbits and was even reversed in mice compared to humans (see Figure 5A). Less pronounced differences between species were seen when comparing anterior with inferior segments (see Figure 5B). Reduced murine radial and long-axis peak velocities could be found for more apical regions in rabbits and humans compared to basal segments as corroborated by the analysis of basal-to-apical velocity ratio in Figure 5C. The comparison between the ratios of different myocardial segments was chosen due to its dimensionless nature, which therefore does not depend on absolute velocity values. The results demonstrate the importance of regional cardiac function, which revealed subtle but significant differences within species. Furthermore, the different rotational behaviour results in a different evolution of twist angles during the cardiac cycle in all three slices in mice compared to humans and rabbits as can be seen in Figure 6.

Our findings have significant implications.

1) Particularly, the observation of a different twist and regionally inhomogeneous motion patterns across species is very important for investigating (transgenic) models of cardiac diseases, where a different twist may lead to different strain patterns and potentially a different remodelling. Our results also show a higher wall thickness to radius ratio in mice, which is potentially relevant for understanding the different rotational patterns across species as speculated by Liu et al. [33]. This is also in line with the findings presented in Figure 7, which illustrates that the endo-to-epi twist ratio is larger in mice, than in the other two species.

2) Pronounced species differences have been reported in cardiac ion channel function, action potential shape [13,14], Ca^2+^ cycling proteins, and electromechanical coupling [4] between rodents and humans. However, it is not clear how these differences impact on myocardial contractile function. Although this question cannot be answered by this study, our results clearly demonstrated that significant differences exist. Rabbits, which have cardiac ion channels [14] and Ca^2+^ cycling properties [4] that are more similar to humans than mice also show a closer similarity in myocardial motion patterns. Consequently, rabbit models might be more suitable to mimic the human phenotype of diseases with electrical and mechanical impairment than mice [36]. Indeed, several transgenic rabbit models of human diseases such as long-QT syndrome and hypertrophic cardiomyopathy have demonstrated pronounced similarities with the human phenotype [5,6]. It might be of further interest to investigate other mammalian hearts such as guinea pigs, rats, pigs or dogs that are also commonly used for the exploration of cardiovascular diseases to complete our understanding of species differences in myocardial function.

3) Besides molecular differences in ion channels and calcium cycling and contractile proteins, structural differences such as the myofiber orientation may contribute to species differences in myocardial function. Streeter et al. described the ventricular myocardium as a continuum in which myofiber orientation varied smoothly across the ventricular wall with a fiber angle variation of up to 180° transmurally [37]. Measurements of myofiber orientation throughout the walls of the RV and LV in different mammalian hearts such as dogs [8], pigs [37], rabbits [9], and humans [7] have shown that there is significant local variation of fiber orientation, particularly at the junctions of the RV and LV free walls and in the interventricular septum, which might explain the regional differences and the different rotational characteristics in our study. Recently, Healy et al. published a study comparing the myofiber structure between mice, rabbit, and sheep using diffusion tensor CMR [10]. Most pronounced (and significant) differences were found between mice and both other species. Notably, an opposite helix angle at the anterior junction of the RV and LV was observed in mice.

One can assume that each excitation should give rise to bi-phasic twisting. However, the missing initial un-twisting in mice might be due to the thin ventricular wall (~1.5 mm) and the short conduction delay between endo- and epicardium (~2 ms) [38] not allowing a staged mechanical activation of initially endo- and later epicardial fibers. If the entire LV wall is activated, epicardial fibers dominate (lager mass fraction) resulting in the observed rotational behavior. This may be further established e.g. by a high-speed video of an isolated beating mouse heart.

However, in order to create a link between fiber structure and myocardial contraction, exhaustive computational models of electrical and mechanical function are necessary [39]. A detailed comparison of such differences with respect to the myocardial motion pattern is warranted to thoroughly explain the observed species differences in myocardial motion patterns.

### Limitations

One limitation is given by the fact that a direct comparison between different species is difficult to perform due to differences in absolute myocardial velocities. Moreover, only the myocardial motion pattern with its velocity amplitudes (or velocity ratios) was investigated. Due to the varying heart rates the velocity time courses were normalized to end-systole in order to maintain characteristic features of the motion pattern, particularly during diastole. To allow a comparison of relative temporal differences (of time-to-peak values), systole and diastole should be normalized to account for different heart rates. Therefore, a retrospectively ECG-triggered sequence would be beneficial to reduce errors due to the normalization of the cardiac cycle. However, the analysis of temporal parameters was beyond the scope of this study.

Anesthesia was necessary for CMR experiments in mice and rabbits but was not used in human subjects. Since anesthesia is known to affect blood pressure, heart rate, and cardiac contractility [40,41], the necessity of anesthesia in animal studies may impact on some aspects of regional and global myocardial mechanical function. However, a recent study systematically assessing hemodynamic effects of various anesthetic drugs in mice and rats demonstrated similar global systolic and diastolic myocardial function under isoflurane or ketamine/xylazine anesthesia as assessed by peak rate of pressure rise (dP/dtmax) and decline (dP/dtmin), stroke work and relaxation time - despite differential effects on blood pressure and heart rate [42]. It is expected that the pronounced species differences in regional myocardial mechanical function between mice and rabbits – particularly those in rotational behavior – are thus not due to different anesthetic regimen; however, the effect of anesthetics cannot be definitely ruled out.

## Conclusions

In conclusion, phase contrast CMR revealed a significantly different myocardial motion pattern in mice and rabbits compared to humans. Especially systolic rotation of rabbits more closely resembled human LV performance, a finding that should be considered when investigating myocardial performance using mouse models. These findings dictate that myocardial motion found in animal models cannot simply be transferred one-to-one to human conditions. A deeper knowledge of mammalian-related differences in myocardial motion may help to select (small) animal models that best mimic physiological and pathological human cardiac function when investigating specific myocardial diseases.

## Competing interests

The authors declare that they have no competing interests.

## Authors’ contributions

BJ is responsible for the study design, the data acquisition in humans, analysis and interpretation of data, and drafted the manuscript. KO performed the data acquisition in rabbits and drafted of the manuscript. ED participated in the murine data acquisition and ~ analysis, drafting of the manuscript and interpretation of results. DF participated in the data analysis, drafting of the manuscript and interpretation of results. MMe carried out the statistical analysis. MMa revised the manuscript critically for important intellectual content. JS carried out the murine data acquisition, participated in the study design design and coordination, and drafting and revising the manuscript. All authors read and approved the final manuscript.
